# Associations of anaemia with blood pressure in women of reproductive age: a cross-sectional study in Johannesburg, South Africa

**DOI:** 10.11604/pamj.2024.48.99.43763

**Published:** 2024-07-12

**Authors:** Xolisa Nxele, Elizabeth Symington

**Affiliations:** 1 School of Public Health, University of the Western Cape, Cape Town, South Africa,; 2 Department of Life and Consumer Sciences, University of South Africa, Johannesburg, South Africa

**Keywords:** Anaemia, HIV, hypertension, South Africa, cardiovascular diseases

## Abstract

**Introduction:**

South Africa has approximately 8.45 million adults living with human immunodeficiency virus (HIV) with women being higher at risk. Anaemia is proportional to HIV severity and a predictor for cardiovascular disease. In this study, we aimed to determine associations between anaemia, HIV, and blood pressure among women of childbearing age in Roodepoort, a suburb within the city of Johannesburg.

**Methods:**

in this cross-sectional study premenopausal women were recruited from a primary healthcare facility, Johannesburg. Socio-demographics, lifestyle behaviours, and medical history, including HIV status, were collected. Anthropometrical measurements and blood pressure (BP) were obtained, and venous blood was drawn to determine hemoglobin (Hb) concentration. Multiple and logistic regression analyses were performed to determine the association between hemoglobin (Hb), HIV and blood pressure (BP).

**Results:**

of 228 women, 72% were pregnant and 22% HIV positive. Pregnant women had lower BP (SPB: 104 ± 11 vs 115 ± 11 mmHg, p<0.001; diastolic BP (DBP): 68 ± 8 vs 80±10 mmHg, p<0.001) compared to non-pregnant women. Hb levels were lower among HIV positive compared to HIV negative participants (11.4 ± 1.6 vs 12.1 ± 1.4 g/dL, p=0.010). More HIV positive women were classified as anaemic (37% vs 16%, p=0.003). In unadjusted multiple linear models, Hb concentration was associated with systolic blood pressure (SBP) (β 1.20 (95% CI, 0.28, 2.33), p=0.013) and DBP (β 1.94 (95% CI, 1.08,2.80) p<0.001), and in unadjusted logistic regression models, women with anaemia had increased odds for hypertension (OR 1.18 (95% CI, 1.20, 2.80), p=0.006). However, in both cases, significance was lost when adjusting for covariates.

**Conclusion:**

the results suggest anaemia may be a risk factor for hypertension and should be investigated in larger, homogenous samples.

## Introduction

Approximately 39 million people are living with the human immunodeficiency virus (HIV) globally, and more than 70% of these occur in sub-Saharan Africa [1]. South Africa (SA) has approximately 8.45 million adults living with HIV [2]. People living with HIV (PLHIV) are highly susceptible to developing haematologic abnormalities and the most common of these are peripheral blood cytopenia. Anaemia is the most common cytopenia in PLHIV accounting for 50% of all cytopenia´s, followed by leukopenia and thrombocytopenia [3]. Anaemia pathogenesis is associated with many causes in HIV patients, including the effects of the infection, which suppress the bone marrow and the immune system thereby decreasing blood cell counts [4]. During anaemia, low levels of both haemoglobin (Hb) and haematocrit lead to a decrease in whole blood viscosity. Reduced whole blood viscosity initiates cardiac enlargement and left ventricular hypertrophy due to volume overload. Thus, anaemia is a risk factor for ventricular remodelling, cardiac dysfunction, and myocardial ischemia [5], and severe anaemia is a risk factor for ventricular dysfunction and heart failure [6]. Furthermore, anaemia is known to exacerbate cardiovascular disease (CVD) outcomes in people with high blood pressure [7]. High blood pressure is the most prevalent modifiable CVD risk factor [8], both among PLHIV and generally healthy individuals. Hypertension accounts for approximately 9 million deaths annually and its prevalence ranges between 17%-40% in developing countries [7].

Women of reproductive age, adolescent girls and pregnant women are highly susceptible to anaemia [9,10]. Alarmingly, this population also has the highest prevalence of HIV in SA (26%) [11]. In pregnancy, the aetiology of anaemia is complex, but apart from micronutrient deficiencies, gestational anaemia is largely caused by haemodilution due to a 25%-80% increase in plasma volume and red blood cell (RBC) volume, often referred to as physiological anaemia. These hemodynamic alterations associated with pregnancy, such as volume expansion and increased cardiac output, are considered normal [12]. Even so, gestational anaemia is associated with poor outcomes, for example, preterm birth, low birth weight, low immunity, and miscarriages [9]. Additionally, women living with HIV are more likely to develop anaemia and are at higher risk of developing cardiovascular events than their HIV-negative counterparts [13].

Considering the complex set of risk factors in pregnant women with HIV, this raises the question of whether anaemia can be considered a driver of CVD in pregnant women with HIV. Could this be different between pregnant and non-pregnant women? Are all the risks and all the drivers different for pregnant and non-pregnant women? Thus, the aim of this study was to determine associations between anaemia, HIV, and blood pressure among women of childbearing age in Roodepoort, a suburb within the city of Johannesburg.

## Methods

**Study design and setting:** this cross-sectional study was conducted among generally healthy pregnant and non-pregnant women, either living with or without HIV and of childbearing age (18 to 49 years of age). Women were recruited between September 2022 and May 2023 from the family planning (for non-pregnant women) and antenatal clinics (for pregnant women) at Discoverers Community Health Centre (DCHC), in Roodepoort, a suburb of Johannesburg.

**Participants:** the women were sampled consecutively as they entered the clinic and enrolled in the cardiovascular, haemostatic and micronutrient status of pregnant women in an urban food environments (CHAMP) study, once consent was provided. The recruited women were able to speak and read a local language. Both HIV-infected and uninfected women were included, however, the following cases were excluded from enrolment: multiple pregnancies, known non-communicable disease (namely high blood pressure, high cholesterol, renal disease, and diabetes), known communicable disease (such as hepatitis, tuberculosis) and those who were diagnosed with a known serious illness like systemic lupus erythematosus, cancer, or psychosis.

**Variables, data sources/measurement, and bias:** the primary outcome measures were systolic blood pressure (SBP), diastolic blood pressure (DBP) and heart rate. Blood pressure and heart rate were taken twice by trained fieldworkers using calibrated equipment (Omron M3W upper arm blood pressure monitor, OMRON Healthcare, Kyoto, Japan) [14]. The participant was in a seated position for 5 minutes before the measurements were obtained from the right arm. The second measurement for blood pressure and heart rate was used for analysis. Prehypertension was defined as SBP ≥120 mmHg and/or DBP ≥80 mmHg [15]. Hypertension was defined as SBP ≥140 mmHg and/or DBP ≥90 mmHg [15].

Haemoglobin (Hb) levels were determined from whole venous blood. Venous blood has been shown to provide more accurate Hb values than capillary blood [16]. Blood was drawn into labelled Ethylenediaminetetraacetic acid (EDTA)-coated evacuated tubes by a phlebotomist and transported in cool storage containers to the laboratory for sample preparation within four hours of blood being drawn. Thereafter, 10 μL of whole blood from EDTA-coated evacuated tubes was used to determine Hb concentrations by transferring blood to a microcuvette through capillary action. Microcuvettes were immediately placed in a calibrated HemoCue Hb meter (Hb 801+, Ängelholm, Sweden). Readings were captured immediately before the next sample was analysed. Since hypoxia (for example, due to smoking and elevated altitude of residence) may increase haemoglobin concentrations, haemoglobin results were adjusted, according to World Health Organization (WHO) recommendations [17], for smoking (-0.3 g/dL) and altitude (-0.5 g/dL) due Johannesburg's altitude at 1700m [18]. Anaemia in pregnancy was defined in two ways to allow for comparison with other studies: 1) <11 g/dL for pregnant women in their first and third trimesters and <10.5 g/dL for the second trimester, 2) <11 g/dL for all pregnant women [17]. Anaemia in non-pregnant women was defined as <12 g/dL [19]. HIV status and treatment were obtained from the women´s medical files as obtained using standard operating procedures in public healthcare in South Africa.

Demographic and socio-economic data were collected using interviewer-administered questionnaires. The data included date and country of birth, marital status, level of education, home language, employment status, social grant recipients and number of household members. Additionally, the living standards data was collected to classify the women according to the Living Standards Measure (LSM) [20], which is used to describe the socioeconomic status of the population. To determine pica, women were asked whether they consumed non-food items, such as soil, charcoal, plastic, or clay, in the past month and how often. In addition, women were asked if they use any nutritional supplements (either obtained from the clinic or purchased from stores). Lastly, maternal tobacco use was determined by asking if the women had smoked or used snuff in the past week.

**Anthropometric measurements:** anthropometrical data was collected from all the participants. Body mass index (BMI) was calculated for non-pregnant women (dividing weight in kg by height in m squared (kg/m^2^) since unadjusted pregnancy weight is not an accurate reflection of nutritional status during the second and third trimester. BMI cut-offs were applied as follows: for the underweight, <18.5 kg/m^2^, normal weight 18.50-24.99 kg/m^2^, overweight ≥ 25 kg/m^2^ and obese ≥ 30 kg/m^2^. Weight (kg) was measured while participants were barefoot and wearing light clothing using a flat, calibrated electronic scale (SECA 813, Seca GmbH & Co. KG, Hamburg, Germany). Height measurements were obtained using a portable stadiometer (SECA 213, Seca GmbH & Co. KG, Hamburg, Germany), on a level surface and braced against a wall. Mid-upper arm circumference (MUAC) measurements were performed on all women using a standard spring-wound, metal tape measure [21]. Briefly, the tape measure was placed on the participant´s non-dominant arm midway between the olecranon of the elbow and the acromion process, and the circumference measured horizontally with the arm was in a relaxed position. MUAC < 23 cm was considered to suggest underweight, while > 33 cm to suggested obesity, and a MUAC of 23-33 cm suggested no risk of malnutrition [21]. All measurements were conducted in duplicate. In the instances when there was a discrepancy in weight measurement of more than 0.1 kg, height more than 5 cm, MUAC of more than 1.5 cm, a third measurement was carried out. Then, an average if the first two or last two measurements, depending on if a third measurement was taken, was utilised for further analysis.

The medical records were used to obtain the gestational age for the pregnant women. If an ultrasonography was conducted and recorded during the first or second trimester, these results were utilised to calculate the gestational age as on the day of data collection. When ultrasonography results were not available, the first day of the last normal menstrual period (LNMP) was used to calculate the gestational age. If no ultrasonography or LNMP data was available, the estimated due date (EDD) provided in the medical records was used to calculate the gestational age.

**Study size:** to determine the achieved statistical power, a post hoc power analysis was conducted using G*Power 3.1.9.4. software [22]. The calculation was based on multiple linear regression analysis (fixed model) with a total of 7 predictors; a small to medium effect size F^2^ of 0.07; probability of error (alpha) of 5%; and a total sample size of 228 participants. The result indicated a power of 95% was achieved.

**Ethical considerations:** ethical approval was obtained from the UNISA Health Research Ethics Committee (2020/CAES_HREC/093), and permission was provided by the Gauteng Department of Health, Johannesburg Health District´s Research Committee (GP_202111_043). This study was performed according to the guidelines in the Declaration of Helsinki. Data collection was conducted after the women provided written informed consent.

**Statistical methods:** all hard copy data were captured in Microsoft Excel (Microsoft Corporation, Washington, USA) and quality assurance was conducted on 20% of all the captured data. Statistical analyses were conducted using SPSS version 28.0.1.1 (SPSS Inc, Chicago, IL, USA). Data were tested for outliers and normality using Q-Q plots and histograms. Normally distributed data are expressed as means and standard deviations (±SD), and non-normally distributed data are expressed as medians (25^th^ percentile; 75^th^ percentile). Descriptive statistics were conducted to describe anthropometrical traits, medical history, supplement use and pica. Independent t-tests for continuous variables and chi-square tests for categorical variables were conducted to compare the socio-demographic and health traits between the pregnant and non-pregnant. For chi-square tests where exact significance was not available, asymptotic significance was reported. To prepare for multiple linear regression, assumptions were tested for homoscedasticity; a linear relationship between predictor variables and dependent variables using scatterplots; and normal distribution of residuals. Multiple linear regression analyses were conducted to determine associations between Hb and cardiovascular assessments (SBP, DBP and HR) with maternal age, pregnancy status, HIV status, MUAC and educational level as covariates in model 2, while model 1 was unadjusted. Additionally, binary logistic regression analyses were conducted to determine associations between maternal anaemia as well as HIV and prehypertension and hypertension in pregnant women with maternal age, pregnancy status, HIV status, MUAC and educational level as covariates in model 2, while model 1 was unadjusted. p<0.05 was considered significant and p between 0.05 - 0.10 as having a tendency toward significance.

## Results

**Figure 1:**
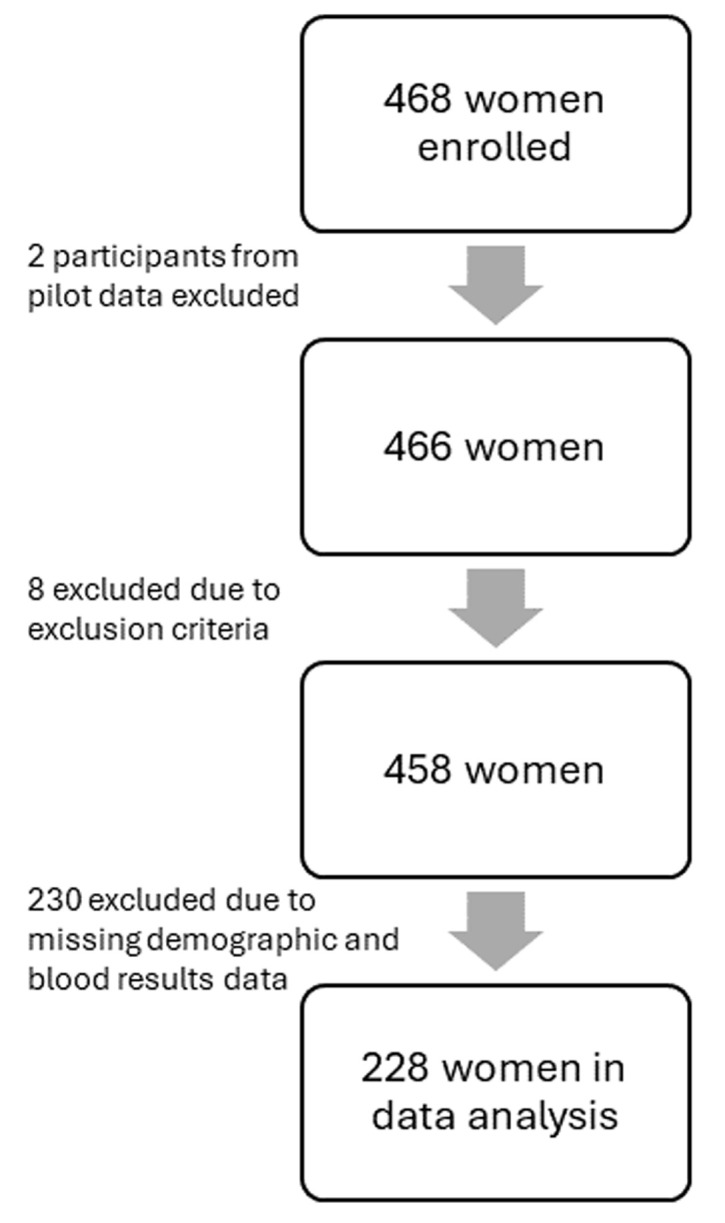
flowchart illustrating study participant enrolment and total number included for analysis

**Participants:** a flow-chart of the women included in the CHAMP study and the availability of data for analysis in this study is shown in Figure 1. A total of 468 participants were enrolled, however, participants who did not meet the inclusion criteria as assessed post-enrolment were excluded (n=8). Additionally, the first two pilot participant data (n=2) were removed, as well as participants who had no Hb results due to stock limitations; and those who did not have any blood drawn and/or questionnaire data (n=230). Therefore, 228 women were included in the data analysis.

**Descriptives:** the participants’ characteristics by total sample and by pregnancy status are shown in Table 1. Demographically, the pregnant women were younger than the non-pregnant women (28.8 ± 6.1 vs 31.2 ± 7.0 years, p=0.013). Most of the sample were South African (65%) and of black ethnicity (91%). Just more than half of the women were living with a partner/married (56%) and most had good education (55% with matric and 23% post-school education). The women had medium to high living standards (72%) with most households having 1 to 5 residents per household (89%). More pregnant women were unemployed (62% vs 43%, p=0.026) and more tended to receive social grants (56% vs 44%, p=0.061) compared to non-pregnant women.

**Table 1:** socio-demographic characteristics and medical history by pregnancy status

Characteristic	Total sample (n=228)	Pregnant (n=165)	Non-pregnant (n=63)	p-value
		n (%)/Mean (±SD)	n (%)/Mean (±SD)	
Age, years	29.47(±6.4)	28.8 (±6.1)	31.2 (±7.0)	0.013
**Marital status**				**0.129**
Married/life partner	127(56)	68(41)	33(52)	
Single	101(44)	97(59)	30(48)	
**Employment**				**0.026**
Unemployed	128(56)	101(62)	27(43)	
Employed	95(42)	61(37)	34(54)	
Student	4(2)	2(1)	2(3)	
Social grant recipient	78(44)	35(56)	28(44)	0.061
**Race**				**0.631**
Black	208(91)	151(92)	57(91)	
Coloured	12(5)	8(5)	4(6)	
Indian	5(2)	3(1)	2(3)	
White	3(1)	3(1)	0(0)	
**Highest level of education**				**0.323**
Post school education	52(23)	33(20)	19(30)	
Grade 12	125(55)	94(57)	31(49)	
Grade 8-11	43(19)	31(19)	12(19)	
Primary school	8(3)	7(4)	1(2)	
**Country of origin**				**0.821**
South Africa	148(65)	108(66)	40(64)	
Zimbabwe	62(27)	45(28)	17(27)	
Malawi	8(4)	5(4)	3(5)	
Lesotho	4(2)	2(1)	2(4)	
Mozambique	2(1)	2(1)	0(0)	
**Number of residents per household**				**0.195**
1 to 5 residents	201(89)	148(90)	53(84)	
6 to 10 residents	26(11)	16(10)	10(16)	
Living Standards Measure (n=227)				0.429
1-4	6(3)	5(3)	1(2)	
5-7	131(58)	98(60)	33(52)	
8-10	90(40)	61(37)	29(46)	
BMI, kg/m^2^ (n=62)		^a^	28.9 (±6.2)	
Underweight (<18.5 kg/m^2^)			0(0)	
Normal weight (18.6-24.9 kg/m^2^)			17(27)	
Overweight (25-29.9 kg/m^2^)			23(37)	
Obese (>30kg/m^2^)			22(36)	
MUAC, cm (n=220)	30(±4.6)	30(±5.0)	32(±4.0)	0.040
<23 cm	7(3)	7(4)	0(0)	
23-33 cm	151(69)	110(70)	41(66)	
>33 cm	62(28)	41(26)	21(34)	
HIV positive (n=209)	46(22)	33(21)	13(26)	0.490
**HIV therapy (n=44)**				**0.301**
TLD	30(68)			
HAART	13(30)			
TEE	1(2)			
Tobacco use (n=227)	19(8)	16(10)	3(5)	0.184
Supplement user (n=222)	164 (74)	160(99)	4(7)	<0.001
Pica (n=220)	78 (36)	65 (41)	13 (21)	0.002
^1^ Anaemic	46(20)	30 (18)	16 (25)	0.225
^2^ Anaemic	61(26)	45 (27)	16(25)	0.775
**Blood pressure**				
Systolic	107(±12)	104(±11)	115(±11)	<0.001
Diastolic	72(±10)	68(±8)	80(±10)	<0.001
Heart rate	81(±12)	83(±11)	77(±14)	<0.001
**Prehypertensive**				
SBP≥120 and/or DBP ≥80	53(24)	21(13)	32(52)	<0.001
**Hypertensive**				
SBP≥140 and/or DBP ≥90	14(6)	2(1)	12(19)	<0.001
Proteinuria		6(4)	^b^	

BMI: Body mass index; MUAC: Mid-upper arm circumference; HIV: human immunodeficiency virus; BP: blood pressure; TEE: Tenofovir disoproxil fumarateemtricitabine-efavirenz; TLD: Tenofovir (TDF) + Lamivudine (3TC) + Dolutegravir (DTG); HAART: highly active antiretroviral therapy; SBP: systolic blood pressure; DBP: diastolic blood pressure; ^a^BMI not provided for pregnant women; independent t-test for continuous variables, chi-square for categorical variables unless otherwise indicated; p < 0.05 to p < 0.1 shows statistical significance; ^b^Proteinuria only assessed in pregnant women; ^1^Anaemia: <11 g/dL for pregnant women in their first and third trimesters and <10.5 g/dL for second trimester; <12 g/dL for non-pregnant women; ^2^Anaemia: <11 g/dL for all pregnant women; <12 g/dL for non-pregnant women

**Outcome data, main results, and other analyses:** in terms of anthropometry, the MUAC of non-pregnant women was larger than those who were pregnant (32 vs 30 cm, p=0.040). Most of the non-pregnant women were either overweight or obese (37% and 36%, respectively). Pica and supplement use were both higher in pregnant women than their non-pregnant counterparts (41% vs 21%, p=0.002 and 99% vs 7%, p<0.001, respectively). Less than a quarter of the women were HIV positive (22%) with no difference in prevalence between pregnant and non-pregnant women (21% vs 26%, p=0.490). There were no differences in the anaemia status between pregnant and non-pregnant women (27% vs 25%, p=0.868). However, blood pressure was lower in pregnant compared to non-pregnant women (SPB: 104±11 vs 115±11 mmHg, p<0.001; DBP: 68±8 vs 80±10 mmHg, p<0.001) and heart rate higher (83±11 vs 77±14 bpm, p<0.001). Similarly, more non-pregnant women were classified as prehypertensive and hypertensive compared to pregnant women (52% vs 13%, p<0.001 and 19% vs 1%, p<0.001, respectively).

Table 2 shows the blood pressure, Hb levels and pica according to the participants’ HIV status. There were no differences in SBP, DBP or HR between HIV-positive and HIV-negative participants. However, Hb levels were lower among HIV-positive participants compared to HIV-negative participants (11.4±1.6 vs 12.1±1.6 g/dL, p=0.010). More HIV-positive women were classified as anaemic (44% vs 23%, p=0.008). When pica was expressed by HIV status, more of the HIV-positive women tended to participate in pica (48% vs 32%, p=0.057). There was no difference in anaemia prevalence between supplement users and non-users (results not shown).

**Table 2:** blood pressure, haemoglobin levels and pica by HIV status

Characteristic	Total group (n=209)	HIV positive (n=46)	HIV negative (n=163)	p-value
	**Mean(±SD)/n (%)**			
SBP	107(±12)	107(±12)	107(±11)	0.470
DBP	71 (±10)	72(±10)	71 (±10)	0.760
HR	81(±11)	82(±13)	81(±11)	0.785
Hb	11.9(±1.5)	11.4(±1.6)	12.1(±1.4)	0.010
^1^ Anaemic	43(21)	17(37)	26(16)	0.003
^2^ Anaemic	57(27)	20(44)	37(23)	0.008
Pica (n=201)	72(36)	22(48)	50(32)	0.057

SBP: systolic blood pressure; DBP: diastolic blood pressure; HR: heart rate; Hb: haemoglobin; Chi-square for categorical variables unless otherwise indicated; p < 0.05 to p < 0.1 shows statistical significance; ^1^anaemia: <11 g/dL for pregnant women in their first and third trimesters and <10.5 g/dL for second trimester; <12 g/dL for non-pregnant women; ^2^anaemia: <11 g/dL for all pregnant women; <12 g/dL for non-pregnant women

In unadjusted models (model 1), Hb levels were associated with SBP (β 1.20 (95% CI, 0.28,2.33), p=0.013) and DBP (β 1.94 (95% CI, 1.08, 2.80) p<0.001) but not HR (Table 3). However, after adjusting for pregnancy, HIV status, maternal age, MUAC and education level, there were no associations between Hb and cardiovascular outcomes. There were no associations between HIV and any of the heart health outcomes in both unadjusted and adjusted models (adjusted for pregnancy status, maternal age, MUAC and educational level).

**Table 3:** associations between maternal Hb, HIV, and blood pressure (multivariable linear regression, β-values, and 95% confidence intervals)

Systolic blood pressure						
	**Model 1**			**Model 2**		
**Predictor**	**B**	**95% CI**	**p-value**	**B**	**95% CI**	**p-value**
Hb	1.20	(0.28,2.33)	0.013	-0.31	(-1.33,0.71)	0.549
HIV	-0.83	(-3.90,3.73)	0.966	1.92	(-1.60,5.41)	0.281
Diastolic blood pressure						
	Model 1			Model 2		
Hb	1.94	(1.08,2.80)	<0.001	0.41	(-0.40,1.21)	0.307
HIV	-0.21	(-3.60,3.16)	0.900	1.10	(-1.70,3.81)	0.441
**Heart rate**						
	**Model 1**			**Model 2**		
Hb	-0.40	(-1.45,0.64)	0.449	0.301	(-0.84,1.51)	0.601
HIV	-0.99	(-4.92,2.94)	0.620	-3.05	(-7.00,1.00)	0.128

HIV: human immunodeficiency virus; Hb: haemoglobin; CI: confidence interval; p < 0.05 to p < 0.1 shows significance; model 1 for Hb level and HIV status were unadjusted; model 2 for Hb levels was adjusted for pregnancy, HIV, maternal age, MUAC, educational level; model 2 for HIV status was adjusted for pregnancy, maternal age, MUAC, educational level

Logistic regression analyses indicated in unadjusted models that women with anaemia and an increased odds for hypertension (Table 4) (OR 1.18 [95% CI, 1.20,2.80], p = 0.006). However, in the adjusted model the association was lost. HIV status was not associated with prehypertension or hypertension.

**Table 4:** associations between maternal Hb, HIV, prehypertension, and hypertension (binary logistic regression, odds ratios and 95% confidence intervals)

Prehypertensive						
	**Model 1**			**Model 2**		
**Predictor**	**OR**	**95% CI**	**p-value**	**OR**	**95% CI**	**p-value**
Anaemia	1.24	(0.55,2.80)	0.602	1.60	(0.57,4.46)	0.374
HIV	0.73	(0.34,1.57)	0.422	0.88	(0.36,2.14)	0.774
**Hypertensive**						
	**Model 1**			**Model 2**		
Anaemia	1.18	(1.20,2.80)	0.006	2.80	(0.30,26.71)	0.379
HIV	0.89	(0.18,4.36)	0.889	0.67	(0.12,3.78)	0.644

OR: Odds ratio; CI: confidence interval; p < 0.05 to p < 0.1 shows significance; model 1 for Hb level and HIV status were unadjusted; model 2 for Hb levels was adjusted for pregnancy, HIV, maternal age, MUAC, educational level; model 2 for HIV status was adjusted for pregnancy, maternal age, MUAC, educational level; anaemia: <11 g/dL for pregnant women in their first and third trimesters and <10.5 g/dL for second trimester; anaemia: <11 g/dL for all pregnant women

## Discussion

From this cross-sectional study in women of childbearing age, we showed that HIV-positive women had lower Hb levels compared to HIV-negative women, but no difference in BP or HR. Since anaemia has been identified as a predictor for cardiovascular disease, we assessed the association between Hb and BP. Unadjusted models indicated that Hb was a predictor for SBP and DBP, however, when adjusting for several confounders including HIV and pregnancy the association was lost. Similarly, anaemia increased the odds for hypertension in unadjusted binary logistic regression models, but not in adjusted models. Ideally, additional analysis on pregnant or non-pregnant women alone might have shed light on the potential association between anaemia and BP. However, due to the small sample size, the statistical power was insufficient to do so.

The non-pregnant women in the present study showed higher systolic and diastolic BP when compared to their pregnant counterparts. Premenopausal women are thought to have a lower cardiovascular risk than their male counterparts (21), however, there are contrasting views on whether women of reproductive age are as susceptible to hypertension as men or not [23,24]. Black South African women were previously shown to have higher BP than their white counterparts when their systolic and diastolic BPs were monitored for 24 hours [25]. Plus, being overweight is associated with hypertension, with a 49% increased risk per five-unit increase in BMI was previously shown [26]. In this study, from a mainly black African sample, we also observed the majority of non-pregnant women to be overweight or obese (37% and 36%, respectively) and 52% to be pre-hypertensive. Importantly, this study did not enrol women with known hypertension, yet 19% of the non-pregnant women were hypertensive when study BP measurements were conducted. In this sample, several known risk factors for hypertension existed which diluted the statistical power to assess the association of anaemia and hypertension.

The pregnant women showed significantly lower BP readings which was expected since BP is typically lower during pregnancy due to progesterone and relaxin. Increased progesterone causes a decrease in arterial BP and vascular resistance by relaxing the smooth muscles [27]. Although the systolic and diastolic BPs of the pregnant women were in their normal ranges, their heart rates were increased. This is consistent with literature where HR is reported to increase by 10-20 bpm throughout normal pregnancy and reaches its peak in the last trimester [28]. These results suggest a healthy increase in HR consistent with increased gestation.

We further demonstrated that HIV-positive women were more likely to be anaemic than their HIV-negative counterparts. This is in line with literature which shows an increase in anaemia as HIV progresses [29]. Additionally, literature has also shown that women of reproductive age are susceptible to anaemia [10]. Concerningly, in this study, there was a high prevalence of anaemia among HIV-positive women (37%), in comparison to the current statistics on [30] women of reproductive age in South Africa (23%). It should be noted that this is a small sample size of 46 women who were HIV-positive.

Previous studies have shown that anaemia [31] and BP [32] are risk factors for CVD in people living with HIV, with hypertension being classified as a CVD risk factor (24) [33]. In the present study, unadjusted regression models showed anaemic women to have increased odds for hypertension, however, pregnancy, body mass and other covariates may have been the main drivers of this association. Additionally, in other studies, anaemia has been linked with adverse CVD outcomes in hypertensive patients [7]. These results suggest a link between anaemia and hypertension, which could lead to other CVD outcomes and should be investigated further in larger homogenous groups.

A limitation of the study includes a limited sampling area in Johannesburg thus results cannot be generalised to other areas. Furthermore, due to the consecutive sampling method, the sample is not representative of the population in the area and no matching of pregnant and non-pregnant women was done which is clear from the participant characteristics. The non-pregnant women were older, more overweight (as shown with MUAC), and with a higher employment rate, which rendered the comparison between pregnant and non-pregnant groups challenging.

## Conclusion

Anaemia and hypertension are known risk factors for CVD complications. This study showed an association between anaemia and BP in women of childbearing age; however, this association was dependent on pregnancy, weight status, age, HIV status and education levels. Although the causes of anaemia are complex, it has been shown to serve as a predictor for several health outcomes. Since assessing anaemia is simple in public health settings, its application as a predictor of CVD outcomes could be useful in the South African context. Further research on larger homogenous groups on the relationship between anaemia and blood pressure in HIV-positive women would be useful.

### What is known about this topic

- *HIV-positive people are now living longer due to antiretroviral therapy; however, with the increased life expectancy, HIV is now a known risk factor for CVD in these individuals;*
- *Anaemia is a risk factor for CVD, and it accelerates CVD outcomes in people with hypertension;*
- *Pregnant women are at higher risk for anaemia*.

### What this study adds

- *Relationships between anaemia and blood pressure were driven by covariables namely pregnancy, HIV, arm circumference and educational level;*
- *Pregnant and non-pregnant women had similar levels of anaemia; however, we confirmed that more HIV women were anaemic compared to HIV-negative women;*
- *Although we excluded women with hypertension, 19% of the non-pregnant sample were found to be hypertensive upon measuring blood pressure, suggesting the need to increase blood pressure screening*.
